# Epistatic Genetic Effects among Alzheimer’s Candidate Genes

**DOI:** 10.1371/journal.pone.0080839

**Published:** 2013-11-18

**Authors:** Timothy J. Hohman, Mary Ellen Koran, Tricia Thornton-Wells, for the Alzheimer's Neuroimaging Initiative

**Affiliations:** Center for Human Genetics and Research, Department of Molecular Physiology & Biophysics, Vanderbilt University School of Medicine, Nashville, Tennessee, United States of America; Oslo University Hospital, Norway

## Abstract

**Background:**

Novel risk variants for late-onset Alzheimer’s disease (AD) have been identified and replicated in genome-wide association studies. Recent work has begun to address the relationship between these risk variants and biomarkers of AD, though results have been mixed. The aim of the current study was to characterize single marker and epistatic genetic effects between the top candidate Single Nucleotide Polymorphisms (SNPs) in relation to amyloid deposition.

**Methods:**

We used a combined dataset across ADNI-1 and ADNI-2, and looked within each dataset separately to validate identified genetic effects. Amyloid was quantified using data acquired by Positron Emission Tomography (PET) with ^18^F-AV-45.

**Results:**

Two SNP-SNP interactions reached significance when correcting for multiple comparisons, *BIN1* (*rs7561528*, rs744373) *x* *PICALM* (*rs7851179*). Carrying the minor allele in *BIN1* was related to higher levels of amyloid deposition, however only in non-carriers of the protective *PICALM* minor allele.

**Conclusions:**

Our results support previous research suggesting these candidate SNPs do not show single marker associations with amyloid pathology. However, we provide evidence for a novel interaction between *PICALM* and *BIN1* in relation to amyloid deposition. Risk related to the *BIN1* minor allele appears to be mitigated in the presence of the *PICALM* protective variant. In that way, variance in amyloid plaque burden can be better classified within the context of a complex genetic background. Efforts to model cumulative risk for AD should explicitly account for this epistatic effect, and future studies should explicitly test for such effects whenever statistically feasible.

## Introduction

 Novel risk variants for late-onset Alzheimer’s disease (AD) have been identified and replicated in recent genome-wide association studies (GWAS)[1–3]. Although these are the genes with largest effects known for AD, the observed effects of the non-*APOE* risk variants are relatively small, with odds ratios of about 1.2. The complex genetic makeup of AD necessitates explorations that go beyond single marker analysis to look for epistatic relationships that might explain some of the missing heritability. Using the information garnered from large scale GWAS analyses and outcome measures derived from biological processes that are more proximal to gene function, we aim to elucidate how some of these risk variants relate to specific aspects of AD etiology. 

Recent work has begun to address the relationship between these risk variants and biomarkers of AD. One study performed fine mapping of *PICALM*, *BIN1*, *CLU*, and *CR1* and found that these genes were not associated with amyloid pathology or tau pathology measured *in vivo* in cerebrospinal fluid (CSF)[4]. Other evidence has suggested *CR1* is involved in amyloid pathology, cognitive decline, and brain atrophy[5,6]. Moreover, recent work has identified an interaction between a single nucleotide polymorphism (SNP) mapped to the *CR1* gene and *APOE* genotype in relation to amyloid pathology measured by Positron Emission Tomography PET[7]. *BIN1* has had similar mixed associations including the null result previously mentioned and other work suggesting it is related to tau pathology[8], perhaps through its role in the immune response system[9]. Yet to date no single analysis of single marker and epistatic relationships between the replicated SNP hits from these four genes has been performed in relation to neuroimaging measures of amyloid deposition. 

 The aim of the current study was to leverage two independent datasets from the Alzheimer’s Disease Neuroimaging Initiative (ADNI) to identify genetic effects and interactions among the known risk SNPs of AD. First, we sought to evaluate the null-effects reported previously between *PICALM, BIN1, CLU, CR1* and amyloid burden using a PET measure of amyloid, rather than the CSF measure used previously[4]. Similar to the CSF measures, amyloid imaging measures have been related to disease onset and progression, have been validated post-mortem, and more recently were included as a biomarker for classifying patients with mild cognitive impairment (MCI) and dementia in research studies[10–13]. Second, we tested for genetic interactions with APOE-ε4 genotype status given the previous reports of APOE-gene interactions that explain some of the variance in AD disease status[14]. Finally, we tested whether SNP-SNP interactions among these risk genes would explain additional variance in amyloid pathology beyond known variance related to age, disease status, and *APOE* genotype. 

## Methods

 Data used in the preparation of this article were obtained from the ADNI database (adni.loni.ucla.edu). The ADNI was launched in 2003 by the National Institute on Aging (NIA), the National Institute of Biomedical Imaging and Bioengineering (NIBIB), the Food and Drug Administration (FDA), private pharmaceutical companies and non-profit organizations, as a $60 million, 5-year public-private partnership. The primary goal of ADNI has been to test whether serial magnetic resonance imaging (MRI), PET, other biological markers, and clinical and neuropsychological assessment can be combined to measure the progression of MCI and early AD. Determination of sensitive and specific markers of very early AD progression is intended to aid researchers and clinicians to develop new treatments and monitor their effectiveness, as well as lessen the time and cost of clinical trials.

The Principal Investigator of this initiative is Michael W. Weiner, MD, VA Medical Center and University of California – San Francisco. ADNI is the result of efforts of many co-investigators from a broad range of academic institutions and private corporations, and subjects have been recruited from over 50 sites across the U.S. and Canada. The initial goal of ADNI was to recruit 800 adults, ages 55 to 90, to participate in the research, approximately 200 cognitively normal older individuals to be followed for 3 years, 400 people with MCI to be followed for 3 years and 200 people with early AD to be followed for 2 years. For up-to-date information, see ww.adni-info.org.

All data were de-identified and all analyses were deemed exempt by the Vanderbilt IRB per 45 CFR 46.101(b).

### Subjects

Demographic data are presented in Table 1. Participants were enrolled based on the criteria outlined in the ADNI protocol (http://www.adni-info.org/Scientists/AboutADNI.aspx) and the ADNI2/ADNI-GO protocols (http://adni.loni.ucla.edu/wp-content/uploads/2008/07/ADNI_Go_Protocol.pdf ; http://adni.loni.ucla.edu/wp-content/uploads/2008/07/ADNI2_Protocol_FINAL_20100917.pdf). For the present project, analyses were restricted to Caucasian subjects for whom we had both genotype and PET data. 

**Table 1:** Demographic Information.

	Baseline Clinical Diagnosis**^a^**		
	Normal Control	Mild Cognitive Impairment	Alzheimer’s Disease
***ADNI-1 Dataset***			
Number of Patients	67	53	40
Number of APOE- ε4 Carriers	14	18	25
Number of Females	31	17	15
Mean Baseline Age *(SD*)	81.06 (5.04)	79.47 (7.41)	76.75 (6.33)
Mean Years of Education *(SD*)	16.22 (2.78)	15.51 (3.21)	16.15 (2.91)
Mean SUVR**^b^** AV-45 *(SD*)	1.07 (0.16)	1.19 (0.25)	1.31 (0.25)
***ADNI-2/GO Dataset***			
Number of Patients	107	239	24
Number of APOE- ε4 Carriers	26	104	17
Number of Females	52	103	9
Mean Baseline Age *(SD*)	74.83 (5.55)	71.82 (7.44)	73.58 (9.78)
Mean Years of Education *(SD*)	16.42 (2.59)	16.04 (2.64)	15.96 (2.71)
Mean SUVR**^b^** AV-45 *(SD*)	1.11 (0.20)	1.19 (0.22)	1.39 (0.21)
***COMBINED DATASET***			
**Number of Patients**	**174**	**292**	**64**
**Number of APOE- ε4 Carriers**	**40**	**122**	**42**
**Number of Females**	**83**	**120**	**24**
**Mean Baseline Age *(SD*)**	**77.23 (6.15)**	**73.21 (7.99)**	**75.56 (7.88)**
**Mean Years of Education *(SD*)**	**16.34 (2.66)**	**15.94 (2.76)**	**16.08 (2.82)**
**Mean SUVR^b^ AV-45 *(SD*)**	**1.09 (0.19)**	**1.19 (0.23)**	**1.34 (0.24)**

^a^ Normal Control subjects had a Mini-Mental Status Examination (MMSE) score between 24 and 30, a Clinical Dementia Rating (CDR) score of 0, and were not depressed (Geriatric Depression Scale score < 6).

Mild Cognitive Impairment subjects had a MMSE score between 24 and 30, objective memory impairment, subjective memory impairment, and a CDR score of 0.5.

Alzheimer’s Disease subjects met clinical criteria for dementia, had an MMSE of between 20 and 26, and had CDR score of .5 or 1.

^b^ SUVR - Standardized uptake value ratio for amyloid tracer

### Genotyping

 In ADNI-1, genotyping was performed using the Illumina Infinium Human-610-Quad BeadChip[15]. In ADNI-2/GO genotyping was performed using the Illumina OmniQuad array[16]. 

Quality control (QC) was performed using PLINK software (version 1.07)[17] excluding SNPs with a genotyping efficiency < 98%, a minor allele frequency of < 1%, or deviation from Hardy-Weinberg Equilibrium (HWE) < 1e^-6^ . We selected the SNPS from *BIN1, PICALM, CR1, CLU, MS4A6A, EPHA1, CD33, CD2AP*, and *ABCA7* that had been replicated in previous GWAS studies and were present in both datasets, which yielded 10 SNPs for analysis (both SNP hits implicated for *BIN1* were included although they are in linkage disequilibrium in 1000 Genomes, r^2^ = 0.759, D` = 0.92). Subjects were excluded if they had a call rate < 98%, if there was a reported versus genetic sex inconsistency, or if relatedness to another sample was established (PI_HAT > 0.5).

### Quantification of Amyloid Deposition

 Amyloid deposition in ADNI-GO and ADNI-2 was quantified using an ^18^F-AV-45 tracer and has been described extensively elsewhere[18].^,^[19] The Mean Standard Uptake Value Ratio (SUVR) measure was calculated across the cingulate (including anterior and posterior regions), frontal, temporal (including middle and lateral regions), and lateral parietal cortices (including the precuneus and supramarginal gyrus), and divided by the reference region (cerebellar grey matter).

### Single Marker Analyses

 Single marker analyses were performed using univariate regression in SAS 9.3 (http://www.sas.com/software/sas9/; PROC GLM). Mean SUVR was set as our quantitative outcome measure, and we used a full additive model for gene effects. Covariates included age at the time of scan, years of education, sex, diagnosis (Normal, MCI, AD), and APOE-ε4 carrier status (carrier v. non-carrier coded 0/1). A correction for multiple comparisons was performed in the combined dataset using the Bonferroni procedure taking linkage between the two *BIN1* SNPs into account.

### APOE- ε4 Interaction Analyses

*APOE*-ε4 interaction analyses were performed using the PROC GLM procedure in SAS. The model was the same as that used in the single marker analyses above, except that we included an SNP x *APOE* interaction term, which was our term of interest. Correction for multiple comparisons was performed on the T-statistic and p-value derived from this model term.

### SNP-SNP Interaction Analysis

 SNP – SNP interaction analyses were performed using the PROC GLM procedure in SAS using the same model as the single marker analyses above. In this case we included two SNP effects and a SNP-SNP interaction term to test for epistatic effects. The SNP – SNP interaction was our term of interest and correction for multiple comparisons was performed on the T-statistic and p-value derived from this model term, correcting for the total number of SNP-SNP comparisons performed taking into account linkage between the two *BIN1* SNPs (36 total independent tests). 

### Posthoc Binary Logistic Regression

 The variable quantifying amyloid load in the current analyses was not normally distributed within or across diagnostic groups. Although linear regression is known to be fairly robust to deviations from normality, we chose to validate our findings using binary logistic regression because the closest approximation of the mean SUVR distribution was bimodal. A binary variable differentiating amyloid positive versus amyloid negative individuals was derived using a previously identified and accepted cut-point of mean SUVR > 1.11[20]. This variable was set as a binary outcome measure in a logistic regression model using the same parameters as those in the original SNP-SNP interaction analysis above. Binary logistic regression was only run as a posthoc examination of the significant interactions identified in the primary analysis.

## Results

### Single Marker Results

 No single marker effects showed a significant association with amyloid pathology in the combined dataset or in either dataset independently (Table 2). Although outside the aim of the current paper, we included the single marker effect of *APOE* in Table 2 as well. As expected, there was a strong association between *APOE* genotype and amyloid deposition within both cohorts (p < 0.0001). The *CLU* SNP (rs11136000) showed a trend toward an association in ADNI-2/GO, but showed no pattern in the ADNI-1 dataset. As previously reported using CSF data, it appears these candidate SNPs do not show independent associations with amyloid pathology. Additional details for each SNP tested can be seen in Table S1.

**Table 2:** Single Marker Effects and APOE Interactions.

***Main Effects***	ADNI1 Dataset		ADNI2/GO Dataset		**Combined Datasets**					
	T ^a^	p-value	T ^a^	p-value	T^a^	**p-value**	**FWE^b^**	**^c^χ^2^**	**p-value**	**FWE^b^**
*CLU* (rs11136000)	0.02	0.987	-1.77	0.078	-1.27	0.206	1.00	1.20	0.273	1.00
*BIN1* (rs744373)	0.18	0.855	-1.23	0.221	-1.14	0.256	1.00	3.23	0.072	0.65
*BIN1* (rs7561528)	-0.11	0.915	-0.75	0.452	-0.90	0.369	1.00	1.57	0.210	1.00
*EPHA1* (rs11767557)	-1.06	0.290	0.10	0.919	-0.79	0.428	1.00	0.51	0.474	1.00
*CD2AP* (rs9296559)	0.34	0.736	-1.07	0.285	-0.66	0.511	1.00	0.11	0.745	1.00
*MS4A6A* (rs610932)	-0.53	0.596	-0.32	0.750	-0.51	0.607	1.00	2.28	0.131	1.00
*CD33* (rs3865444)	0.34	0.734	0.24	0.809	0.27	0.787	1.00	0.00	0.951	1.00
*CR1* (rs3818361)	-1.08	0.283	0.40	0.692	-0.23	0.815	1.00	0.01	0.919	1.00
*PICALM* (rs3851179)	-0.04	0.970	0.13	0.894	-0.04	0.966	1.00	0.48	0.487	1.00
*ABCA7* (rs3764650)	1.93	0.055	-1.22	0.222	0.02	0.987	1.00	0.07	0.784	1.00
***APOE (binary)***	**5.86**	**<0.0001**	**9.95**	**<0.0001**	**11.46**	**<0.0001**	**<0.0001**	**75.86**	**<0.0001**	**<0.0001**
***APOE Interactions***	ADNI1 Dataset		ADNI2/GO		**Combined Dataset**					
	T ^d^	p-value	T ^d^	p-value	**T^d^**	**p-value**	**FWE^b^**	**^e^χ^2^**	**p-value**	**FWE^b^**
***BIN1* (rs744373)**	0.83	0.410	**2.07**	**0.039**	**2.11**	**0.035**	0.31	3.23	0.072	0.65
*BIN1* (rs7561528)	-0.16	0.872	2.32	0.021	1.73	0.084	0.75	2.66	0.103	0.92
*MS4A6A* (rs610932)	-0.75	0.456	-1.07	0.284	-1.46	0.145	1.00	0.86	0.354	1.00
*CD2AP* (rs9296559)	-0.35	0.725	-1.00	0.317	-1.16	0.247	1.00	1.23	0.267	1.00
*CD33* (rs3865444)	2.41	0.017	-0.37	0.714	.97	0.332	1.00	0.02	0.902	1.00
*PICALM* (rs3851179)	1.06	0.290	-1.62	0.107	-.78	0.439	1.00	1.18	0.278	1.00
*CLU* (rs11136000)	-0.72	0.475	1.23	0.218	.66	0.508	1.00	0.81	0.367	1.00
*ABCA7* (rs3764650)	1.29	0.200	0.11	0.913	.59	0.552	1.00	1.15	0.284	1.00
*EPHA1* (rs11767557)	-0.31	0.756	0.77	0.442	.39	0.697	1.00	1.15	0.283	1.00
*CR1* (rs3818361)	0.53	0.594	-0.32	0.753	.01	0.991	1.00	0.02	0.901	1.00

^a^ T value for SNP model term

^b^ FWE: Bonferroni correction for multiple comparisons taking into account linkage between the two *BIN1* SNPs (9 independent tests)

^c^ χ^2^ for SNP term in the binary logistic regression model using amyloid positivity as outcome measure

^d^ T value for SNP x *APOE* interaction term

^e^ χ^2^ for SNP x *APOE* interaction term in the binary logistic regression model using amyloid positivity as outcome measure

### APOE Interaction Results

 In the APOE interaction analyses, the *BIN1* x *APOE* interaction showed nominal significance in the combined dataset, T (521) = 2.09, p = 0.037, but was not significant when correcting for multiple comparisons. This trend was only present in the individuals from the ADNI-2/GO cohort. *CD33* (rs3865444) showed a nominal association in ADNI-1, T(521) = 2.41, p = 0.017, but this effect was not present in ADNI-2/GO. 

### SNP-SNP Interaction Results

**Figure 1:**
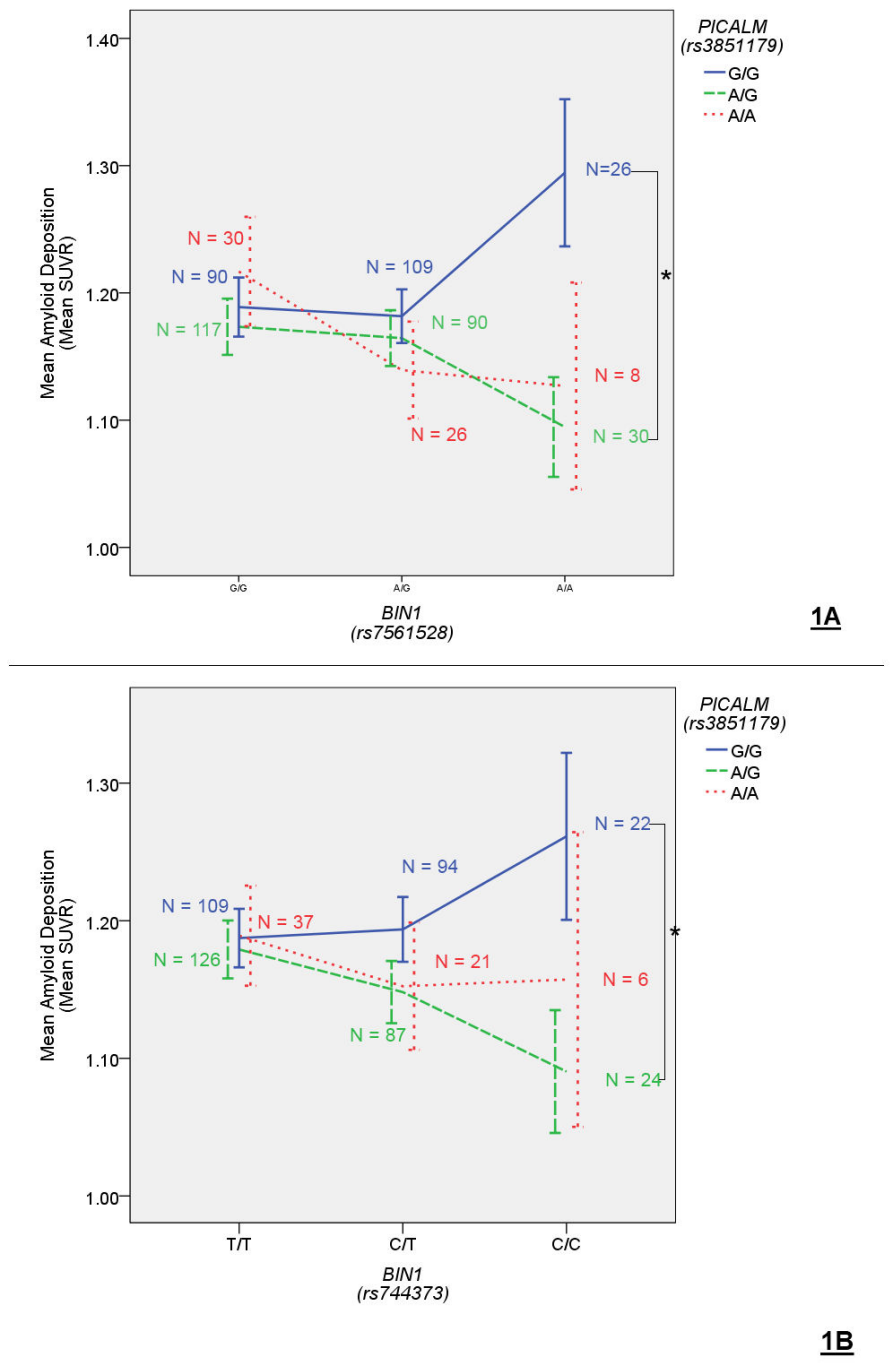
PICALM *x* BIN1 and amyloid deposition. The top two interactions were rs3851179 at the PICALM locus with rs7561528 (Figure 1A) and with rs744373 (Figure 1B) at the BIN1 locus. Error bars represent standard error. *p < 0.05 (two-tailed).

**Figure 2:**
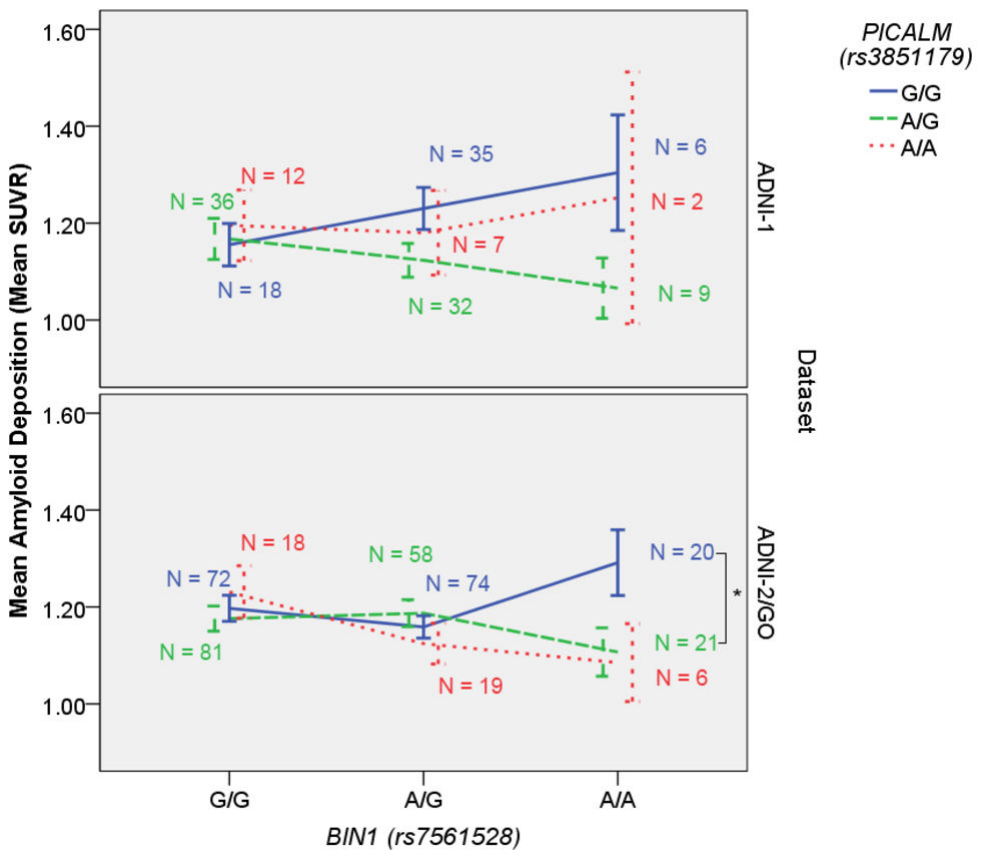
PICALM *x* BIN1 Interaction Across Datasets. The strongest interaction is graphed across the two independent datasets. The top panel displays subjects genotyped on the ADNI-2/GO chip and the bottom panel displays subjects genotyped on the ADNI-1 chip. Error bars represent standard error. *p < 0.05 (two-tailed).

 In the SNP – SNP interaction analyses, five SNP x SNP interactions showed an association with amyloid deposition (Table 3). Of these, only the *BIN1* (rs7561528) x *PICALM* (rs3851179) remained statistically significant when correcting for multiple comparison taking linkage into account. The other *BIN1* SNP (rs744373) also interacted with *PICALM* although the effect did not reach statistical significance (Figure 1). Among the two data sources, the rs7561528 x rs3851179 interaction showed a significant association in the ADNI-2/GO dataset alone and showed a trend in the ADNI-1 dataset (Figure 2). The other nominally significant interactions included *CD2AP* (rs9296559) x *PICALM* (rs3851179), *CLU* (rs11136000) x *PICALM* (rs3851179), and *CLU* (rs11136000) x *MS4A6A* (rs610932). In all cases the effects were only observed in ADNI-2/GO dataset. It appears that, although these candidate genes do not show main effects on amyloid pathology, genetic interactions between them do explain additional variance in plaque burden quantified using PET. 

**Table 3:** SNP-SNP Interaction Analysis.

***SNP – SNP Interactions***	ADNI-1 Dataset		ADNI2/GO Dataset		**Combined Datasets**					
	T^a^	p-value	T^a^	p-value	**T^a^**	**p-value**	**FWE^b^**	**^*c*^χ^2^**	**p-value**	**FWE^b^**
***BIN1* (rs7561528) x *PICALM* (rs3851179)**	-1.89	.061	**-2.83**	**0.005**	**-3.25**	**0.0012**	**0.04**	**7.89**	**0.0050**	0.18
*BIN1* (rs744373) x *PICALM* (rs3851179)	-1.57	0.118	**-2.31**	**0.021**	**-2.71**	**0.0070**	0.25	**5.01**	**0.0252**	0.90
*CD2AP* (rs9296559) x *PICALM* (rs3851179)	0.42	0.672	**2.60**	**0.010**	**2.51**	**0.0125**	0.45	1.83	0.176	1.00
*CLU* (rs11136000) x *PICALM* (rs3851179)	-1.37	0.174	**-2.23**	**0.026**	**-2.36**	**0.0186**	0.67	2.30	0.129	1.00
*CLU* (rs11136000) x *MS4A6A* (rs610932)	0.60	0.550	**2.40**	**0.017**	**2.10**	**0.0361**	1.00	2.98	0.084	1.00

^a^ T value for SNP x SNP interaction term

^b^ FWE: Bonferroni correction for multiple comparisons taking into account linkage between the two *BIN1* SNPs (36 independent tests)

^c^ χ^2^ for SNP x SNP interaction term in the binary logistic regression model using amyloid positivity as outcome measure

## Discussion

 This project investigated single marker and epistatic effects among candidate SNPs for AD in relation to amyloid deposition. We added additional support for the lack of single marker associations between these SNPs and amyloid deposition; however, we provide new evidence for an epistatic relationship between *BIN1* x *PICALM*. This interaction suggests that using powerful quantitative endophenotypes derived from neuroimaging measures may be a fruitful avenue for exploring epistatic relationships among candidate genes in order to explain some of the missing heritability of AD. 

BIN1 x PICALM

 The *BIN1* x *PICALM* interaction appears to explain an additional 1% of the variance (R^2^_full – reduced_ = 0.014) in amyloid load and is biologically plausible given the role these genes play in the process of clathrin-mediated endocytosis[9]. *PICALM* in particular has been suggested to be involved in Aβ clearance[21] and amyloid pathogensis through its role in regulating Aβ metabolism[22]. The minor allele of the replicated *PICALM* candidate SNP used in these analyses (rs3851179) is related to a decreased risk of Alzheimer’s disease (OR = 0.89)[14], suggesting that the minor allele of this SNP should be related to decreased amyloid burden. The observed interaction effect is in line with such an expectation, as minor allele carriers of this rs3851179 showed lower levels of amyloid deposition than non-carriers, but the effect was only present in those individuals also carrying the *BIN1* risk allele. Thus, the protective effect of *PICALM* appears to be relegated to those individuals who carry some other genetic risk factor for AD pathology. 

 Recent work has suggested that while both *BIN1* and *PICALM* both play a role in calthrin-mediated endocytosis (as previously described), only *PICALM* is related to the secretion of the Aβ peptide[23]. The previous null association between *PICALM* and Aβ[4], and the current null result in our single marker analyses suggest that the relationship between *PICALM* and Aβ may be more complex. Indeed, the interaction effect we observe suggests that variation in *PICALM* and *BIN1* combine to modify risk for amyloid deposition. Additional work aimed at analyzing the functional relationship between *BIN1*, *PICALM*, and Aβ may help clarify the mechanisms of this observed statistical interaction. 

### Strengths and Limitations

The present results must be interpreted within the framework of our statistical models. In all cases, we included covariates related to disease status and progression including age, diagnosis, and *APOE* carrier status. Thus, significant interactions are explaining variance beyond known predictors of risk, and while the contributions of these interactions appear to be meaningful, the implications should not be extended without considering the variance accounted for by the other factors in our model. We provide some suggestions as to the functional significance of the SNPs identified, but future work specifically relating these SNPs to gene expression and Aβ secretion may help clarify these genetic interactions.

The difference in effects between ADNI-1 and ADNI-2/GO could be attributed in part to sample size, and the additional subjects in ADNI-2/GO likely provided the additional power necessary to detect interaction effects. However, the two cohorts also differed in their distribution of clinical status, with a greater percentage of AD cases and a much lower percentage of MCI patients in ADNI-1. Although the distribution of mean SUVR values did not significantly differ between the two cohorts (t(546) = 1.28, p = 0.771), we cannot rule out the possibility that the difference in diagnostic category had some effect on our result. For that reason, we also repeated tests for all significant interactions including a cohort covariate. The *BIN1 x APOE* interaction was unchanged (p = 0.035) and the two *BIN1 x PICALM* interactions were more significant (p = 0.0009 and p = 0.006 respectively). Ultimately, additional analyses in a cohort with a more comparable clinical picture to that of ADNI-2/GO may be useful in further validating our results.

To reduce the total number of comparisons, we chose to focus in on the SNPs which had shown a replicated effect on AD rather than looking across the gene. Certainly the previously identified genetic effects are expected to act at the gene level[24], and thus other SNP-SNP interactions between these genes may be able to explain the same or additional variance. Future work approaching epistatic relationships from the gene or pathway level may help clarify the observed interaction and provide additional targets for future functional analyses. 

## Supporting Information

Table S1 **Genotypes for Candidate SNPs Stratified by Diagnostic Category.**
(DOCX)
