# Supplementary material for: Epistatic Genetic Effects among Alzheimer’s Candidate Genes
Source: PLoS One. 2013 Nov 18;8(11):e80839. doi: 10.1371/journal.pone.0080839 (PMC3832488; doi:10.1371/journal.pone.0080839)
Supplement: Table S1 — Genotypes for Candidate SNPs Stratified by Diagnostic Category. (DOCX) [file pone.0080839.s001.docx]

|  |  |  | Diagnostic Category | | | |
| --- | --- | --- | --- | --- | --- | --- |
| ***SNP*** | χ^2^ | p-value^a^ | Normal Control | Mild Cognitive Impairment | Alzheimer’s Disease | |
| *CLU* (rs11136000) | 0.24 | 0.624^a^ |  | | |  |
| C/C |  |  | 60 | 98 | 24 | |
| C/T or T/C |  |  | 87 | 159 | 30 | |
| T/T |  |  | 27 | 34 | 10 | |
| *BIN1 (*rs744373 | 0.33 | 0.563 |  |  |  |  |
| T/T |  |  | 90 | 149 | 33 | |
| T/C or C/T |  |  | 66 | 108 | 28 | |
| C/C |  |  | 16 | 34 | 2 | |
| *BIN1* (rs7561528 | 0.02 | 0.867 |  |  |  |  |
| G/G |  |  | 81 | 129 | 27 | |
| A/G or G/A |  |  | 73 | 122 | 30 | |
| A/A |  |  | 19 | 39 | 6 | |
| *EPHA1* (rs11767557) | 2.61 | 0.106 |  |  |  |  |
| T/T |  |  | 115 | 206 | 35 | |
| C/T or T/C |  |  | 49 | 79 | 25 | |
| C/C |  |  | 9 | 7 | 4 | |
| *CD2AP* (rs9296559) | 0.09 | 0.755 |  |  |  |  |
| T/T |  |  | 93 | 152 | 34 | |
| C/T or T/C |  |  | 73 | 123 | 25 | |
| C/C |  |  | 8 | 17 | 5 | |
| *MS4A6A* (rs610932) | 0.65 | 0.420 |  |  |  |  |
| C/C |  |  | 49 | 115 | 20 | |
| A/C or C/A |  |  | 85 | 137 | 31 | |
| A/A |  |  | 40 | 39 | 12 | |
| *CD33* (rs3865444) | 0.13 | 0.717 |  |  |  |  |
| G/G |  |  | 88 | 140 | 28 | |
| T/G or G/T |  |  | 69 | 124 | 31 | |
| T/T |  |  | 17 | 27 | 4 | |
| *CR1* (rs3818361) | 2.25 | 0.134 |  |  |  |  |
| C/C |  |  | 124 | 192 | 41 | |
| T/C or C/T |  |  | 47 | 91 | 20 | |
| T/T |  |  | 3 | 9 | 3 | |
| *PICALM* (rs3851179) | 1.39 | 0.238 |  |  |  |  |
| G/G |  |  | 70 | 130 | 27 | |
| A/G or G/A |  |  | 80 | 125 | 34 | |
| A/A |  |  | 24 | 37 | 3 | |
| *ABCA7* (rs3764650) | 1.50 | 0.221 |  |  |  |  |
| T/T |  |  | 148 | 234 | 58 | |
| G/T or T/G |  |  | 24 | 53 | 6 | |
| G/G |  |  | 2 | 5 | 0 | |

^a^ χ^2^ represents additive effect of SNP using a binary disease status (AD v. Control) as outcome
